# Personalized Medicine’s Bottleneck: Diagnostic Test Evidence and Reimbursement

**DOI:** 10.3390/jpm4020163

**Published:** 2014-04-04

**Authors:** Joshua P. Cohen, Abigail E. Felix

**Affiliations:** Tufts Center for the Study of Drug Development (Tufts CSDD), Tufts University Medical School, 75 Kneeland Street, Suite 1100, Boston, MA 02111, USA; E-Mail: abigail.felix@tufts.edu

**Keywords:** Personalized medicine, companion diagnostics, reimbursement, cost-effectiveness, coverage with evidence development

## Abstract

Background: Personalized medicine is gradually emerging as a transformative field. Thus far, seven co-developed drug-diagnostic combinations have been approved and several dozen *post-hoc* drug-diagnostic combinations (diagnostic approved after the drug). However, barriers remain, particularly with respect to reimbursement. Purpose, methods: This study analyzes barriers facing uptake of drug-diagnostic combinations. We examine Medicare reimbursement in the U.S. of 10 drug-diagnostic combinations on the basis of a formulary review and a survey. Findings: We found that payers reimburse all 10 drugs, but with variable and relatively high patient co-insurance, as well as imposition of formulary restrictions. Payer reimbursement of companion diagnostics is limited and highly variable. In addition, we found that the body of evidence on the clinical- and cost-effectiveness of therapeutics is thin and even less robust for diagnostics. Conclusions, discussion: The high cost of personalized therapeutics and dearth of evidence concerning the comparative clinical effectiveness of drug-diagnostic combinations appear to contribute to high patient cost sharing, imposition of formulary restrictions, and limited and variable reimbursement of companion diagnostics. Our findings point to the need to increase the evidence base supportive of establishing linkage between diagnostic testing and positive health outcomes.

## 1. Introduction

The Food and Drug Administration (FDA) has stated that the era of one-size-fits-all medicine may be over as pharmaceutical companies increasingly adopt approaches to drug development which involve the use of biomarkers to stratify patient populations [1]. Biomarkers are benchmarks that can be measured to indicate the presence or absence of a disease, or the likelihood of developing a disease. For example, blood glucose levels are a biomarker for diabetes, and blood cholesterol levels are a biomarker for heart disease. Diagnostic tests are generally used to identify the presence, absence, or amount of a biomarker. Companion diagnostics are molecular tests that stratify a patient population with regard to the likelihood of response to, or the safety of, a pharmaceutical therapy. As such, they are critical building blocks in personalized medicine.

The FDA 2011 draft guidance on companion diagnostics provides information on regulatory pathways for drug-diagnostic combinations [2]. Companion diagnostics can be co-developed with a therapeutic, or developed after a drug has been approved. We refer to this as *post-hoc*. Alternatively, an existing test used in conjunction with a drug can be repurposed for a different drug. At a societal level, population stratification offers the potential for a more efficient drug development process. It also may confer more optimal use of society’s health care resources, as targeted care will be more clinically effective, with fewer complications and adverse events.

The drug and diagnostic pipeline is rich with products in development in Phases II and III. Additionally, the Food and Drug Administration currently lists 112 approved products with pharmacogenomics information on the label [3]. Three dozen of these products have information on the label which either suggests or requires use of a companion diagnostic. Information on the label states the likelihood of benefit, possibility of genetic links to side effects, and ways to optimize dosing. To date, there have been seven co-developed therapeutics and diagnostics: Trastuzumab/HER-2 (1998), Crizotinib/ALK (2011), Vemurafenib/BRAF (2011), Ivacaftor/G551D (2012), Trametinib/BRAF (2013), Dabrafenib/BRAF (2013), and Afatinib/EGFR (2013) [4,5]. In addition, there are dozens of *post-hoc* approved personalized medicines across multiple therapeutic categories, including cancer, autoimmune disorders, and cardiovascular disease.

However, there are challenges with obtaining reimbursement of drugs and diagnostics. In this study, we analyze barriers with respect to reimbursement of personalized drugs and their companion diagnostics. Specifically, we analyze formulary management of 10 high-profile drug-diagnostic combinations by payers managing Medicare Parts B (physician-administered) and D (outpatient). Section 2 describes the methods we employ to conduct our study. In Section 3, we present the results. Section 4 concludes with a discussion of our findings as well as certain policy implications.

## 2. Methods

To analyze reimbursement barriers facing uptake of personalized medicine we examined Medicare coverage of 10 drug-diagnostic combinations. We chose to examine Medicare reimbursement because Medicare beneficiaries are disproportionately impacted by personalized medicine, as many of the conditions they suffer from—e.g., cancer, rheumatoid arthritis—have a significant pharmacogenomic component. Moreover, Medicare payers play a key role in driving reimbursement decisions in non-commercial and commercial insurance markets.

We selected 10 drugs and their companion diagnostics for our analysis. The 10 drug-diagnostic combinations were selected based on their global market impact (*i.e*., sales). We collected global sales data for the 10 drugs, as well as U.S. data on annual cost per patient. Our sales and annual cost per patient data sources included Thomson Reuters Cortellis, Medicare Part B pricing files, and the Medicare Part D Formulary Finder. In addition, we collected data from the Tufts Medical Center on the price of each companion diagnostic. Note, there is no publicly available information on sales of companion diagnostics.

Our analysis of formulary management of drug-diagnostic combinations in the U.S. comprised the 20 leading Medicare Part D (outpatient drugs) plans in terms of numbers of covered lives, and 20 Medicare Part B (physician-administered drugs) contractors. For each plan and contractor we examined coverage, patient cost-sharing, and formulary restrictions on the formulary. Medicare Part D plans feature a three-tier formulary, augmented by a fourth or fifth specialty tier designated primarily for biologics and injectables. Formulary restrictions include prior authorization, quantity limits, and step edits. The latter refers to reimbursement of a more costly medication on the formulary only after a less costly alternative has been tried. Part B contractors do not maintain formularies, but periodically publish local coverage determinations (LCDs) to establish whether a drug or diagnostic is “reasonable and necessary” and therefore reimbursable. LCDs are often used to define the appropriate use of certain new medical technologies (usually high volume, high cost).

To further investigate coverage of companion diagnostics, payer views on diagnostic accuracy and clinical utility (*i.e*., linkage between diagnostic usage and positive health outcomes, or net benefit to patients), and the current coding system for diagnostics, we developed a survey instrument. See Appendix. We sent the survey to 20 Part D plans (same subset as the formulary review) and 20 Part B contractors.

Finally, we reviewed the Tufts cost-effectiveness registry to retrieve peer-reviewed publications on the clinical- and cost-effectiveness of the 10 drug-diagnostic combinations. The registry is a comprehensive database of published cost-utility analyses. We used each of the 10 drugs and diagnostics/biomarkers as keywords in a registry search for U.S.-based clinical- and cost-effectiveness studies.

## 3. Results

Three of the drug-diagnostic combinations were co-developed while seven were *post-hoc*. See Table 1. Three are physician-administered (Part B), and seven self-administered (Part D). Looking at aggregated, global sales and annual U.S. costs per patient of the 10 drugs we observe that five out of 10 are considered blockbusters (over $1 billion in annual sales). See Table 2. The weighted growth in sales from 2011 to 2012 was approximately 10%, with projected growth from 2012 to 2013 of around 5%. The most recently approved products on this list—vemurafenib and crizotinib—have relatively low global sales in 2011 and 2012, due in part to not having been approved in Europe until February and November 2012, respectively.

**Table 1:** 10 Drug-diagnostic combinations.

Brand-Name (generic)/Indication	Test(s)/Biomarker(s)	Co-Developed	Medicare Part B or Part D
Herceptin (trastuzumab)—breast cancer	HER-2/neu receptor	Yes	Part B
Gleevec (imatinib)—chronic myeloid leukemia	Philadelphia chromosome/BCR-ABL	No	Part D
Erbitux (cetuximab)—colorectal cancer	EGFR expression/K-RAS mutation	No	Part B
Tarceva (erlotinib)—non-small cell lung cancer	Cobas EGFR mutation	No	Part D
Sprycel (dasatinib)—chronic myeloid leukemia	Philadelphia chromosome/BCR-ABL	No	Part D
Vectibix (panitumumab)—colorectal cancer	EGFR expression/K-RAS mutation	No	Part B
Tykerb (lapatinib)—breast cancer	HER-2/neu receptor	No	Part D
Selzentry (maraviroc)—HIV	CCR5 receptor	No	Part D
Zelboraf (vemurafenib)—non-small cell lung cancer	Cobas BRAF V600E	Yes	Part D
Xalkori (crizotinib)—melanoma	ALK	Yes	Part D

We examine reimbursement of 10 drug-diagnostic combinations. Three of the drug-diagnostic combinations were co-developed while seven were *post-hoc*. Three are physician-administered (Medicare Part B), and seven self-administered (Medicare Part D).

**Table 2:** Global sales of drugs and annual cost per patient (U.S.).

Drug Name (Generic)	Companion Diagnostic/Biomarker	U.S. Approval Date	2013 Global Forecast (in millions of U.S. $)	2012 Global Sales (in millions of U.S. $)	2011 Global Sales (in millions of U.S. $)	2012 Annual Cost per U.S. Patient
Herceptin (trastuzumab)	HER-2/neu receptor	9/25/1998	6,589	6,282	5,944	$47,000
Gleevec (imatinib)	Philadelphiachromosome/BCR-ABL	5/10/2001	4,618	4,675	4,659	$76,000
Erbitux (cetuximab)	EGFR expression/ K-RAS mutation	2/12/2004	1,839	1,843	1,881	$80,000
Tarceva (erlotinib)	Cobas EGFR mutation	11/18/2004	1,439	1,402	1,415	$73,000
Sprycel(dasatinib)	Philadelphiachromosome/BCR-ABL	6/28/2006	1,257	1,019	803	$123,000
Vectibix (panitumumab)	EGFR expression/ K-RAS mutation	9/27/2006	589	954	539	$52,000
Tykerb (lapatinib)	HER-2/neu receptor	3/13/2007	335	379	102	$67,000
Selzentry (maraviroc)	CCR5 receptor	8/6/2007	231	203	176	$15,000
Zelboraf (vemurafenib)	BRAF V600E	9/17/2011	389	250	35	$78,000
Xalkori (crizotinib)	ALK	8/26/2011	282	123	16	$149,000

Sources: Thomson Reuters Cortellis [6]; DataRx [7]; Medicare Part B pricing files [8]; Medicare Part D Formulary Finder [9]. Aggregated global sales are not broken down by indication. Trastuzumab, imatinib, and cetuximab have multiple indications. Therefore, the reported figures reflect sales across all indications for these three drugs. To estimate annual costs per patient, we used information on standard dosing or treatment cycle and extrapolated for one year of treatment for a typical patient.

### 3.1. Drug Reimbursement

All drugs are reimbursed by all Part D plans and Part B contractors. In addition, all drugs are subject to formulary restrictions—prior authorization, quantity limits, or step edits—by at least one payer. On average, each payer imposed formulary restrictions on 85% of the 10 drugs. All drugs are subject to patient cost-sharing that is designated in terms of co-insurance. This implies patients must pay a percentage of the drug’s cost out-of-pocket, as opposed to fixed co-payments. All Part B drugs have patient co-insurance of 20%. And, 85% of Part D drugs are placed in the highest cost-sharing tier; Tier 4. The average patient co-insurance in Tier 4 is 30%, with a range from 10% to 40%.

### 3.2. Diagnostic Reimbursement

Seven of the 8 diagnostics are FDA-approved; and seven out of 8 have analyte-specific codes. See Table 3. The term “analyte-specific” indicates that the code used for reimbursement claims refers to the test’s active ingredient. Officially, companion diagnostics, like physician-administered drugs, are reimbursed under the Medicare Part B medical benefit. Medicare Part B contractors have discretion to determine whether a drug or diagnostic is “reasonable and necessary” and therefore reimbursable.

**Table 3:** Diagnostics/biomarkers.

Diagnostic/Biomarker (drug)	U.S. Price per Test	FDA-Approved	Analyte-Specific Coding
**HER-2/neu receptor (trastuzumab, lapatinib)**	$300	Yes	Yes
**EGFR expression (cetuximab, panitumumab)**	$300	No	Yes
**K-RAS mutation (cetuximab, panitumumab)**	$450	Yes	Yes
**CCR5 receptor (maraviroc)**	$2000	No	Yes
**Philadelphia Chromosome/BCR-ABL (imatinib, dasatinib)**	$200	Yes	No
**ALK (crizotinib)**	$1500	Yes	Yes
**BRAF V600E mutation (vemurafenib)**	$500	Yes	Yes
**Cobas EGFR mutation (erlotinib)**	$1500	Yes	Yes

Sources: Tufts Medical Center; Clarient, [10]; Dako, [11]; Integrated Oncology: LabCorp Specialty Testing Group; [12]; Food and Drug Administration Medical Devices, [13]. The BCR-ABL and K-RAS biomarker tests are used in conjunction with two drugs: cetuximab and panitumumab. Only one of the 8 diagnostic tests did not have analyte-specific coding.

We found that three of 20 Medicare Part B contractors issued nine local coverage determinations (LCDs) related to the 10 drug-diagnostic combinations. Three of the 10 drugs were included in LCDs. Seven LCDs evaluated drug-diagnostic combinations, while two only reviewed diagnostic tests in general terms. The seven LCDs on drug-diagnostic combinations recommended diagnostic testing prior to reimbursement of the drug. The two LCDs that solely focused on diagnostic testing recommended reimbursement of diagnostic tests listed in the right-hand column in Table 4.

**Table 4:** Medicare Part B local coverage determinations (LCDs).

Drug, Documentation on Testing (Number of LCDs)	Drug Approved for Reimbursement	Diagnostic/Biomarker(s) Associated with Drug
*Trastuzumab (2x)*	Yes (on-label)	HER-2
*Cetuximab (3x)*	Yes (on- and off-label)	K-RAS
*Vemurafenib (2x)*	Yes (on-label only)	K-RAS
*Diagnostic testing (1x)*	Yes, all tests for biomarkers listed in right-hand column	K-RAS, BRAF, ALK, HER-2
*Biomarkers for oncology (1x)*	Yes, all tests for biomarkers listed in right-hand column	K-RAS, BRAF, ALK, HER-2

Source: Centers for Medicare and Medicaid Services [14]. We found 9 local coverage determinations (LCDs) published by three Medicare Part B contractors. Three of the drugs in our sample were included in seven local coverage determinations. The seven LCDs on drug-diagnostic combinations recommended diagnostic testing for reimbursement. Additionally, we found two LCDs that evaluated and recommended diagnostic testing in general terms.

Our search of the Tufts cost-effectiveness registry—using the generic names of the 10 drugs in our analysis as keywords, as well as all associated diagnostics/biomarkers—retrieved 16 peer-reviewed studies. Six of the personalized drugs were included in clinical- and cost-effectiveness studies in the Tufts registry. Diagnostics were included in four of the 16 clinical- and cost-effectiveness studies. See Table 5.

**Table 5:** Tufts cost-effectiveness registry analysis.

Brand (generic)	Indication	Number of cost-effectiveness studies	Test included in cost-effectiveness study	Considered cost-effectiveness
Herceptin (trastuzumab)	Breast cancer	8	Yes, 2 of 8	6 of 8
Gleevec (imatinib)	Chronic myeloid leukemia	4	No	2 of 4
Erbitux (cetuximab)	Colorectal cancer	1	Yes	Yes
Tarceva (erlotinib)	Non-small cell lung cancer	1	Yes	Inconclusive
Sprycel (dasatinib)	Chronic myeloid leukemia	1	No	Yes
Vectibix (panitumumab)	Colorectal cancer	0	No	N.A.
Tykerb (lapatinib)	Breast cancer	1	No	No
Selzentry (maraviroc)	HIV	0	No	N.A.
Xalkori (crizotinib)	Non-small cell lung cancer	0	No	N.A.
Zelboraf (vemurafenib)	Melanoma	0	No	N.A.

Source: Tufts cost-effectiveness registry [15]. We found a total of 16 U.S.-based studies in the registry. Four studies included a companion diagnostic in the analysis. There were no clinical- or cost-effectiveness studies for four of the 10 drugs in our sample.

**Figure 1:**
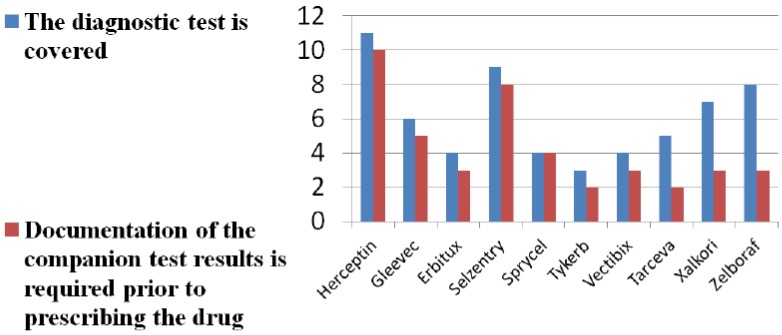
Diagnostic test coverage and documentation.

**Figure 2:**
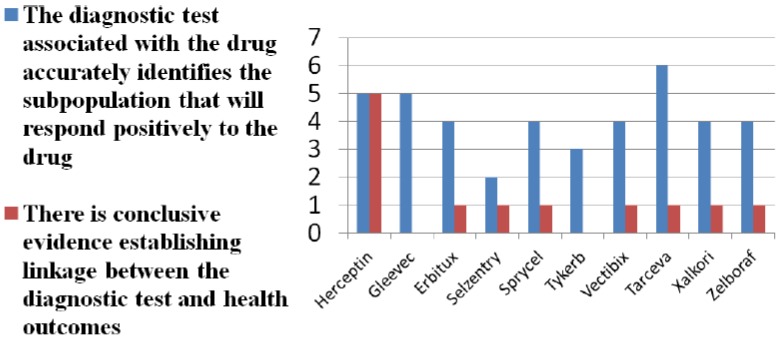
Do tests matter?

Eleven of 40 Medicare payers and contractors returned completed surveys on companion diagnostic reimbursement (26% response rate). Nine represented Part D plans and two Part B contractors. Our survey findings show limited and variable reimbursement of companion diagnostics for all new patients being prescribed the companion drug. See Figure 1. A majority of survey respondents cite lack of evidence on diagnostic accuracy of tests. See Figure 2. A larger majority question the clinical utility of tests. Payer skepticism regarding the diagnostic accuracy and utility of tests is further reflected in the fact that only a minority of payers require documentation that a diagnostic has been conducted prior to prescribing and reimbursement, even in cases in which the diagnostic is on the label and recommended or required by FDA.

Completed survey questionnaires were filled out by 11 payer respondents. We asked payers to report on whether they: (a) reimburse diagnostic tests for all new patients being prescribed the drug in question; and (b) whether documentation of the companion test results is required prior to prescribing the drug.

Completed survey questionnaires were filled out by 11 payer respondents. We asked payers to report on the diagnostic accuracy of the test(s) associated with the drug and the clinical utility of the test(s). The latter refers to linkage between the test(s) and positive health outcomes.

All survey respondents considered clinical effectiveness and health outcomes in their assessment of drug-diagnostic combinations for reimbursement purposes. Three out of 11 considered the cost of a drug per patient, and five assessed cost of a diagnostic per patient. Seven considered the total cost across all patients expected to be prescribed the drug or eligible to take the diagnostic test. And, 10 out of 11 considered the current coding system for reimbursement unsatisfactory. Current Procedural Terminology (CPT) codes for companion diagnostics are developed by the American Medical Association and implemented by the Centers for Medicare and Medicaid Services (CMS). At present, in many instances, CPT codes for companion diagnostics are not analyte-specific and merely reflect technical laboratory processes or activities, such as assay assessment. To build an improved evidence base with respect to establishing the clinical utility of individual companion diagnostics, 9 survey respondents said they would adopt coverage with evidence development policies. Coverage with evidence development implies providing patient access to newly launched diagnostics while evidence is being generated to determined real-world effectiveness. In addition, three survey respondents would implement a tiered formulary for diagnostics. A tiered formulary for diagnostics implies placing relatively costly tests with limited market experience in higher tiers while more information is gathered on the tests’ evidence, and placing tests that have proven value in lower tiers. 

## 4. Discussion and Conclusions

From our study, it appears that all payers reimburse personalized drugs, but with relatively high and variable patient cost-sharing, in addition to imposition of formulary restrictions. Diagnostic reimbursement is limited and variable. Ideally, reimbursement policies governing companion diagnostics should depend on coverage guidelines in place for the accompanying drugs. In other words, a personalized drug should not be reimbursed without an appropriately defined or identified sub-population. However, we observe that drugs are being reimbursed independent of companion diagnostic coverage. When drug and diagnostic are tied in from the outset—co-development—this increases the likelihood of therapeutic success and improved cost-effectiveness. Reimbursement decisions for both the drug and diagnostic would appear to be relatively straightforward, as clinical utility in the sub-population delineated by the diagnostic is supposedly demonstrated. Hence, drug reimbursement would imply diagnostic reimbursement. Nevertheless, we observe that even in cases of co-developed combinations drug reimbursement does not necessarily imply diagnostic reimbursement.

Limited and variable reimbursement may be due to lack of evidence of the tests’ clinical utility [16,17,18]. As we see in our study, there is little published evidence on the clinical- and cost-effectiveness of drug-diagnostic combinations, and very few published local coverage determinations, too. Moreover, in cases in which there is evidence, limited and variable reimbursement may be due to lack of an association between diagnostic testing and positive health outcomes. Several studies have demonstrated that use of a diagnostic test has not mattered with regard to producing better health outcomes [17,18,19].

A clear timeline and process exist for the review of newly approved drugs on a periodic basis by payer Pharmacy & Therapeutics Committees. By contrast, no such formal process exists for the evaluation and coverage of diagnostic tests [20]. Also, there is no standardized method of preparing evidence of clinical utility, establishing whether a diagnostic is covered, or setting reimbursement rates for companion diagnostics. Furthermore, decision-making regarding test coverage is not transparent. A Medicare Part B contractor was quoted as saying: “There are currently no standardized thresholds or benchmarks for evaluating the medical necessity of emerging biomarkers.” And, despite being “aware that there are numerous potentially medically reasonable and necessary therapy-directing genetic tests, many questions remain, among them the lack of—and difficulty in establishing—literature support of medical necessity; lack of standardized testing protocols; lack of robust data for establishing patient-selection criteria; absence of analyte-specific coding” [21].

Important changes are underway supportive of establishing an evidence base for the use of companion diagnostics. First, the Current Procedural Terminology (CPT) coding system has recently been overhauled, with hundreds of new codes being created for companion diagnostics [22,23]. Assignment of analyte-specific codes is needed to facilitate reimbursement, as such codes better reflect the diagnostic’s value [24,25]. By 2014, individual plans or contractors are to assign each test an analyte-specific code, analogous to ICD-9 codes for drugs. In turn, each code is to be priced by individual contractors in accordance with the “value” of the test. In November 2011, Palmetto, a Medicare Part B contractor, became the first to institute a new payment system that assigns a unique code to companion diagnostics [26,27,28]. Under Palmetto’s payment system, called the Molecular Diagnostic Services Program (MolDx), applicants must demonstrate diagnostic tests make a difference, that is, improve patient outcomes and change physician behavior with respect to management of the patient.

Second, some payers have begun asking drug and diagnostic manufacturers to be engaged in Phases II and III, where they can have an impact on clinical trial design end-points [29,30]. To illustrate, in a publicly disclosed partnership, the pharmacy benefit manager Express Scripts and Sanofi are working together to identify biomarkers to guide patient recruitment, collect data on proposed endpoints and comparators for trials, and conduct post-marketing trials for certain (undisclosed) Sanofi products. Pharmaceutical manufacturers are now “backing up to Phase III and asking … what payers would like to know about a drug [and its companion diagnostic], while providing outcomes and endpoints, a drug’s effect on quality of life, and results using a control rather than a placebo.” 

Third, certain payers are implementing coverage with evidence development (CED) policies for which lack clear evidence regarding their clinical utility [30]. CED links Medicare reimbursement of specific promising technologies, such as companion diagnostics, to a requirement that the patients participate in a registry or post-marketing clinical trial. At the time of a diagnostic’s launch the clinical and economic benefits may not be known with great confidence [31]. In such instances, CED would allow access to the companion diagnostic provided evidence is generated in real-world conditions so that payers are better informed to make reimbursement decisions [32,33]. 

## Supplementary Files

Supplementary File 1 Supplementary Materials (PDF, 381 KB)
