# Supplementary material for: Personalized Medicine’s Bottleneck: Diagnostic Test Evidence and Reimbursement
Source: J Pers Med. 2014 Apr 4;4(2):163–75. doi: 10.3390/jpm4020163 (PMC4263971; doi:10.3390/jpm4020163)
Supplement: Supplementary File 1 — Supplementary Materials (PDF, 381 KB) [file jpm-04-00163-s001.pdf]

Supplementary Material

Payer Survey

Q1 1. Please complete the table below:

	Does the diagnostic test associated with the drug accurately identify the subpopulation that will respond positively to the drug?	Is there conclusive evidence establishing a link between the diagnostic test and positive health outcomes?	Do you require documentation that a diagnostic test has been conducted, and that there is a particular test result prior to a drug's reimbursement?	Do you currently provide reimbursement for the companion diagnostic associated with each of the drugs listed in the far left column?
Herceptin-trastuzumab (HER2/ neu receptor)	❑	❑	❑	❑
Erbitux-cetuximab (EGFR expression/ K-RAS test	❑	❑	❑	❑
Gleevec-imatinib (c-kit/PDGFRA)	❑	❑	❑	❑
Selzentry-maraviroc (CCR5 receptor)	❑	❑	❑	❑
Sprycel-dasatinib (Philadelphia Chromosome/BCR-ABL)	❑	❑	❑	❑
Tarceva-erlotinib (cobas EGFR mutation test)	❑	❑	❑	❑
Tykerb-lapatinib (HER-2/neu receptor)	❑	❑	❑	❑
Vectibix-panitumumab (EGFR expression/ K-RAS test)	❑	❑	❑	❑
Xalkori-crizotinib (ALK)	❑	❑	❑	❑
Zelboraf-vemurafenib (BRAF 400E)	❑	❑	❑	❑

Q2 2. In evaluating personalized medicines, to what degree do you consider the following?

	Do not consider	Somewhat consider	Strongly consider
Clinical effectiveness	❑	❑	❑
Health outcomes	❑	❑	❑
Cost of the drug per patient	❑	❑	❑
Total cost across all patients who are prescribed the medicine (*i.e.*, budget impact)	❑	❑	❑

Q3 3. In evaluating diagnostic tests associated with personalized medicines, to what degree do you consider the following?

	Do not consider	Somewhat consider	Strongly consider
Clinical utility (*i.e.*, ability to accurately stratify subpopulations)	❑	❑	❑
Health outcomes	❑	❑	❑
Cost of the diagnostic test	❑	❑	❑
Total cost of diagnostic tests if taken by all who could take the test	❑	❑	❑

Q4 4. As a payer should you be permitted to limit the coverage of certain drugs to patients whose diagnostic test results indicate they are likely to benefit from those drugs?

❍ Yes
❍ No

Q5 5. The current coding system for reimbursement of diagnostic tests is satisfactory (reflects the value of the diagnostic):

❍ Agree
❍ Disagree

Q6 6. Which of the following policies would you employ to improve the reimbursement system for companion diagnostics (you may choose more than one):

❑ Coverage with Evidence Development: Provide access to newly launched diagnostics while evidence is being generated to determine real-world effectiveness.

❑ Tiered Formulary for Diagnostics: Place expensive new tests with limited market experience in higher tiers while more information is gathered on the tests' evidence. Place tests that have proven their value in lower tiers.

❑ Free-Market Pricing with Test-Specific Codes: Manufacturer sets the price for each diagnostic. A test-specific code is granted (similar to the system in place for drugs and biologics). Payers would choose whether to reimburse each test.

Q7 7. On the following scale, please mark the relative importance of factors that may influence the reimbursement of personalized medicines and their companion diagnostics:

	Not Significant	Significant	Very Significant
Scientific establishment of a link between test results and drug response	❑	❑	❑
Ease of testing	❑	❑	❑
Cost of testing	❑	❑	❑
Whether diagnostic is FDA-approved or not	❑	❑	❑
